# A Seasonal Autoregressive Integrated Moving Average (SARIMA) forecasting model to predict the epidemic trends of scrub typhus in China

**DOI:** 10.1371/journal.pone.0325905

**Published:** 2025-06-23

**Authors:** Pei-Ying Peng, Hui-Ying Duan, Lei Xu, Ji-Qin Sun, Li-Juan Ma, Ya Zu, Ting-Liang Yan

**Affiliations:** 1 Institute of Microbiology of Qujing Medical College, Qujing, Yunnan Province, China; 2 Department of Clinical Laboratory, Qujing Second People’s Hospital, Qujing, Yunnan Province, China; Kalinga Institute of Medical Sciences, INDIA

## Abstract

Scrub typhus is a deadly infectious disease that is frequently underdiagnosed. Forecasting the emergence of infectious diseases using epidemiological models has emerged as a crucial instrument for comprehending the dynamics of their occurrence. This research aimed to investigate epidemic traits and create a predictive model for scrub typhus in mainland China, employing the Seasonal Autoregressive Integrated Moving Average (SARIMA) time series method. Monthly records of scrub typhus cases were gathered from the China Center for Disease Control and Prevention, covering the timeframe from 2006 to 2019. From 2006 to 2018, a total of 142849 scrub typhus cases were reported in China, the females’ morbidity was higher than the males’ one (*P* < 0.001). The ideal model was SARIMA (1, 0, 2) (1, 1, 1) _12_ with its residual being white noise (*P* > 0.05). This method forecasted scrub typhus cases between January and December 2019, with the predicted values for 2019 falling within the 95% confidence range. The research indicates that the SARIMA model accurately simulated the epidemiological patterns of scrub typhus across mainland China. Utilizing the SARIMA model is a practical approach for tracking scrub typhus cases in mainland China.

## Introduction

Scrub typhus is a deadly infectious disease that is frequently underdiagnosed. It is spread by feeding on larval chigger mites and is caused by the obligatory intracytosolic bacterium, *Orientia tsutsugamushi* [1]. The clinical manifestations of scrub typhus were characterized by fever, typical eschar or rash accompanied by lymphadenectasis and could be life-threatening due to multiple organ failure [2]. Over 1 billion people are thought to be residing in endemic regions at the moment, and 1 million cases are reported each year [3,4]. Previously, it was thought to be endemic in Asia, Australia, and islands in the Pacific and Indian oceans; this region was known as the Tsutsugamushi Triangle [5–7]. Not only is scrub typhus not restricted to the Tsutsugamushi Triangle, but it can also be caused by orientiae other than *Orientia tsutsugamushi*, as demonstrated by an increasing amount of serological, molecular, genetic, and culture data [8–10].

Scrub typhus was first recognized in China in 1948, but it remained isolated to the tropics and subtropics of southern China for a long time [11]. Only in the last three decades has the disease spread to northern China, including Shandong, Jiangsu, and Anhui provinces [12]. Scrub typhus has become more common in a region of China that is countrywide, according to earlier research [12,13]. Scrub typhus has spread widely and grown to be a serious health issue in China in recent decades [13,14]. Scrub typhus cases have dramatically increased in both frequency and geographic distribution, which may indicate that this neglected tropical illness is making a resurgence [15].

Optimizing the distribution of medical resources and services, exploring the temporal dynamic changes of transmission and prevalence, and forecasting future incidence should be our top priorities in response to the health problems linked to scrub typhus infection. In order to do this, scrub typhus occurrence must be predicted using statistical and mathematical models as an early warning system. The auto-regressive integrated moving average (ARIMA) model is widely used for forecasting the prevalence of epidemic diseases such as scrub typhus. This system is used to simulate a time series’ temporal dependence structure, accounting for periodic variations, random disturbances, and shifting trends [16,17]. SARIMA (Seasonal autoregressive integrated moving average) is a more advanced form of the ARIMA model that requires seasonality, periodicity, and randomness. It takes into account both the overall trend and seasonal variance, as was common in time series models [18]. Because scrub typhus has regular seasonal characteristics, a SARIMA model may be more useful than an ARIMA model in forecasting the scrub typhus outbreak. However, no research has focused on predicting scrub typhus epidemics using particular seasonal characteristics found in China. As a result, the goal of this work was to create a prediction model for scrub typhus in China utilizing the SARIMA algorithm.

## Materials and methods

### Ethics statement

It was determined by the National Health and Family Planning Commission, China, that the data collection for natural-focal diseases cases was part of the continuing public health surveillance system of notifiable infectious diseases in China and was exempt from the institutional review board assessment (Natural-focal diseases are a large group of diseases. At present, there are more than 180 kinds of natural-focal diseases in the world, including scrub typhus).

No work with human subjects was directly involved in our study. Scrub typhus data were extracted from Chinese National Communicable Disease Surveillance Network. All patient records were anonymized and de-identified prior to analysis.

### Data collection and management

**Fig 1:**
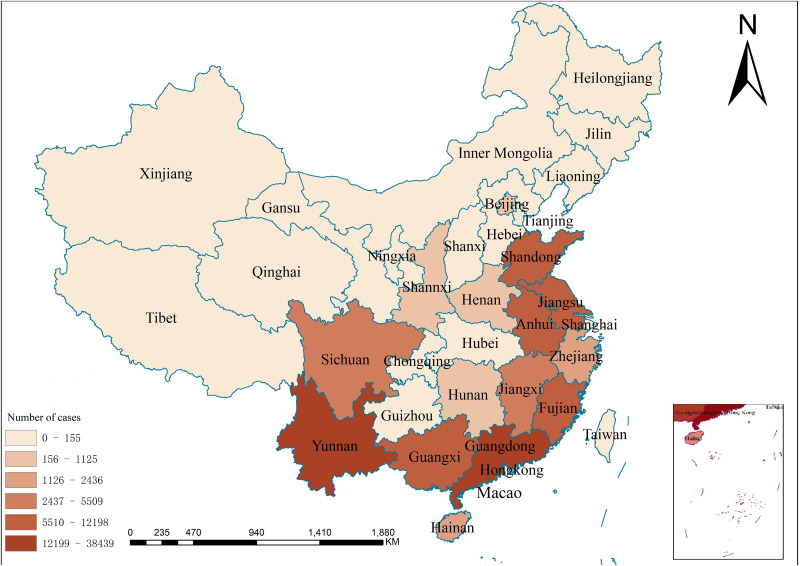
A map of the location of provinces in China. Color-coded regions are cases of scrub typhus in the top 8 provinces, China, 2006–2018 (Dark colors indicate a high number of scrub typhus cases and light colors indicate a low number of scrub typhus cases). **This map was created by ArcGIS software (version 10.6, ESRI Inc.; Redlands, CA, USA). Homepage of ArcGIS software was** https://www.esri.com/.

Scrub typhus is a vector-borne notifiable infectious disease. Every medical institution was required to report to the Chinese Center for Disease Control and Prevention through China’s National Statutory Infectious Disease Reporting Information System (CNNDS) (http://www.chinacdc.cn/, accessed on 10 March 2021). The reported information includes age, occupation, onset, and date of onset and diagnosis, case category, and residential address, etc. For this study, the data from the scrub typhus cases in China from January 2006 to December 2018 were extracted from CNNDS, with a total number of 142849 from 2006 to 2018. Individual-level data on human cases had been de-identified to protect patient confidentiality. Monthly cases of scrub typhus reported at the province level during 2006–2018 were obtained. Time-series analysis for scrub typhus cases were carried out with IBM SPSS Statistics version 27.0 (IBM Corp., NY, USA). Spatial distribution analyses of scrub typhus every province was conducted using Spatial Mapping in ArcGIS version 10.6 (Fig 1). The Chinese Center for Disease Control and Prevention’s national guidelines serve as the basis for diagnosing scrub typhus (http://www.chinacdc.cn/tzgg/200901/t20090105_40316.htm). Weil-Felix OX_*K*_ agglutination titer≥1:160, clinical manifestations (such as high fever, skin rash, lymphadenopathy, and eschars or ulcers), and epidemiological exposure histories (visiting an endemic area and coming into contact with chiggers or rodents within three weeks prior to the onset of illness) are the criteria for a probable case of scrub typhus. A confirmed case of scrub typhus must meet the above criteria for a probable case and at least one laboratory criterion for confirmatory diagnosis: either isolate *Orientia tsutsugamushi* from clinical specimens, detect *O. tsutsugamushi* by polymerase chain reaction (PCR) in clinical specimens, or have a fourfold or greater increase in serum IgG antibody titers between acute and convalescent sera as determined by indirect immunofluorescence antibody assay (IFA) [19].

### SARIMA model

Box and Jenkins introduced the seasonal auto-regressive integrated moving average (SARIMA) model as a time series forecasting technique in the 1970s [20]. The SARIMA model’s general form is as follows: (*p*, *d*, *q*) (*P*, *D*, *Q*) _*S*_, where *d* is the order of the differences, *p* and *q* are the orders of auto-regressive (*AR*) and moving average (*MA*), respectively. *P*, *D*, and *Q* are the corresponding seasonal orders, along with *S* is the steps of the seasonal differences (*S* = 12 for monthly data), which can be computed using the following formulas [21]:


$$\phi (B)\Phi \left(B^{S}\right){\left(1-B^{S}\right)}^{D}(1-B{)}^{d}z_{t}=\theta (B)\Theta (B^{S})\varepsilon_{t}$$

With


$$\phi (B)=1-\phi_{1}B-\ldots -\phi_{p}B^{p}$$

$$\theta (B)=1-\theta_{1}B-\ldots -\theta_{q}B^{q}$$

$$\Phi \left(B^{S}\right)=1-\Phi_{1}B^{S}-\ldots -\Phi_{P}B^{PS}$$

$$\Theta \left(B^{S}\right)=1-\Theta_{1}B^{S}-\ldots -\Theta_{Q}B^{QS}$$

Where *ε*_*t*_ is the residual error at time *t*, *Z*_*t*_ is the observed value at the time *t* (*t* = 1, 2,... *k*), and *B* is the backward shift operator.

By employing monthly scrub typhus case data from January 2006 to December 2018 and a scrub typhus case forecast from January 2019 to December 2019, a SARIMA (*p*, *d*, *q*) (*P*, *D*, *Q*) _12_ model was built using the procedures listed below. Firstly, pre-treatment of the sample. Building a SARIMA model requires no white noise, stationarity, and seasonality in a time series. A correlation between observed values with a non-random distribution and a time series with no white noise can be utilized to construct a model. To ascertain whether the time series is stationary, the augmented Dickey-Fuller (ADF) test was used, which is the most popular unit root testing technique. The EViews program was used to conduct the ADF test. It may be possible to eliminate this trend with differencing if the time series is non-stationary. The seasonality of the time series can be studied using the sequence diagrams and practical knowledge. Secondly, autocorrelation function (ACF) and partial autocorrelation function (PACF) plots were used to identify and estimate the model. The *p*, *q*, *P*, *Q*, and candidate SARIMA models’ model parameters were established based on this [22]. Thirdly, parameters and white noise tests were needed to confirm the model’s availability. To ascertain if the residuals were independently or normally distributed, the Ljung-Box *Q* test was employed. To ascertain whether the model parameters were statistically significant, a t-test and a *P* value were employed. The model parameters’ statistical significance was determined using a *t*-test and a *P* value. Finally, we determined the most optimal model to forecast future scrub typhus epidemic trends by utilizing the lowest Bayesian information criterion of Schwarz (BIC) values, the highest *R*^2^ values, and their residual white noise (*P* > 0.05). White noise is a pure random sequence in time series, which has the characteristics of stationary disorder and no autocorrelation. Therefore, white noise sequence itself does not have useful information value. In general, before time series modeling, it is necessary to confirm that the variables are non-white noise; after modeling, it is necessary to test that the residual sequence is white noise to ensure that all useful information of the residual is extracted. Scrub typhus cases were predicted using the selected SARIMA model between January and December of 2019. The prediction was verified using the 2019 scrub typhus cases that were reported.

### Accuracy test

A prospective prediction was made using the optimal model, and the model’s accuracy was evaluated using the mean absolute percent error (MAPE) and root mean squared error (RMSE).

### Statistical analysis

The differences of morbidity rates and the incidence rates of scrub typhus cases by sex, geographic areas were analyzed using the chi-square test with SPSS v. 27.0 (IBM Corp., NY, USA). *P* values < 0.05 were considered statistically significant. SARIMA modeling and predictions were done using EViews for Windows, version 8.0 (IHS Global Inc., Irvine, CA, USA).

## Result

### Epidemiological feature analysis

From 2006 to 2018, there were 142849 cases of scrub typhus documented in mainland China, including 69 deaths. The yearly morbidity has increased by 20 times, from 0.09/10 0000 in 2006 to 1.93/10 0000 in 2018 (S1 Table). The prevalent seasons were summer and autumn-winter, with the majority of cases falling between July and November. The morbidity rate for females was greater than that of males (*P* value calculated by use of χ^2^ test, *P* < 0.001) (S3 Table). Guangdong, Yunnan, Anhui, Guangxi, Fujian, Jiangsu, Shandong, and Jiangxi were the eight provinces from which the largest number of cases (91.82%) were reported (Tables 1–3, Fig 1).

**Table 1:** Epidemiologic features of scrub typhus cases in China, 2006 −2018.

Year	Cases	Incidence rate/100,000	Deaths	Mortality rate/100,000
2006	1244	0.09	0	0
2007	1332	0.10	1	0.0001
2008	2592	0.19	3	0.0002
2009	3235	0.24	0	0
2010	4085	0.30	11	0.0008
2011	6020	0.45	6	0.0004
2012	8921	0.66	9	0.0007
2013	11104	0.82	4	0.0003
2014	16032	1.19	8	0.0006
2015	17280	1.28	3	0.0002
2016	21691	1.60	12	0.0009
2017	22556	1.62	6	0.0004
2018 Total	26757 142849	1.93 10.66	6 69	0.0004 0.0052

**Table 2:** Reported cases and annual average incidence of scrub typhus in 31 provinces, China, 2006–2018.

Province	Total number of cases	Annual average incidence rate/100,000
Guangdong	38439	2.8344
Yunnan	35420	5.9274
Anhui	12198	1.5770
Guangxi	11718	1.9585
Fujian	9935	2.0714
Jiangsu	9299	0.9094
Shandong	8642	0.6940
Jiangxi	5509	0.9508
Sichuan	4053	0.3877
Hainan	2436	2.1609
Zhejiang	1758	0.2485
Hunan	1125	0.1318
Beijing	914	0.3585
Shaanxi	436	0.0898
Henan	434	0.0355
Hubei	155	0.0208
Guizhou	111	0.0246
Liaoning	71	0.0125
Chongqing	59	0.0157
Tianjing	39	0.0232
Helongjiang	33	0.0066
Heibei	18	0.0019
Gansu	11	0.0033
Shanxi	9	0.0019
Inner Mongolia	8	0.0025
Jilin	6	0.0017
Tibet	5	0.0128
Xinjiang	4	0.0014
Shanghai	2	0.0007
Qinghai	1	0.0014
Ningxia	1	0.0012

**Table 3:** Sex, age and geographic area characteristics description of scrub typhus cases in China, 2006 −2018.

Parameters	Cases	Incidence per 100,000	Proportion	Male% vs. Female%	*P* value
**Sex**					< 0.05^*^
Female	76368	11.70	53.46		
Male	66481	9.68	46.54		
Sex ratio	0.87				
**Age**					
0-9	11702	7.99	8.19	58:42	
10-19	4962	2.84	3.47	64:36	
20-29	7593	3.32	5.32	52:48	
30-39	12811	5.95	8.97	53:47	
40-49	24753	10.75	17.33	47:53	
50-59	32784	20.48	22.95	42:58	
60-69	29609	29.67	20.73	42:58	
≥70	18635	26.55	13.05	42:58	
**Geographic area** ^**#**^					<0.001^*^
Rural	123789	18.36	88.00	43:57	
Urban	17090	2.57	12.00	52:48	

* *P* value calculated by use of χ^2^ test.

# Two columns (Rural and Urban) do not add up to equal the total because of missing data.

### SARIMA model

**Fig 2:**
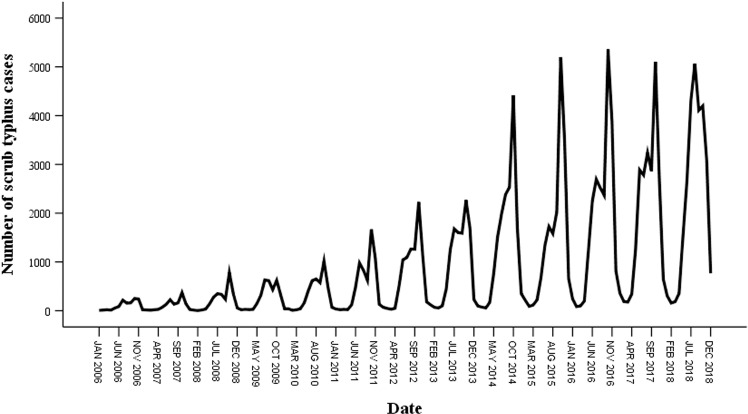
Sequence map of monthly number of scrub typhus cases in China from 2006 to 2018.

**Fig 3:**
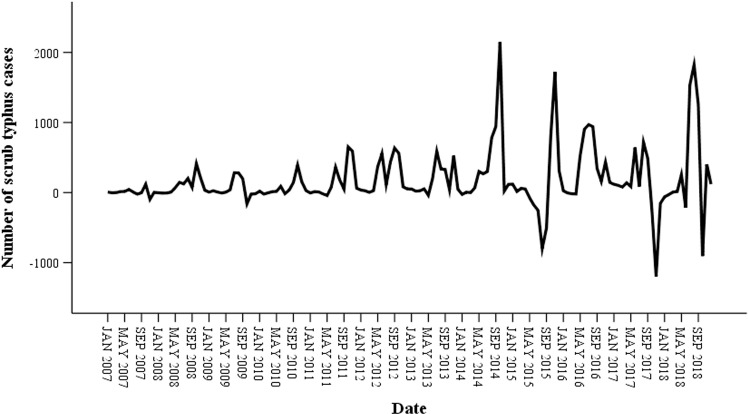
Sequence map of monthly number of scrub typhus cases after first-order seasonal difference with one-period of 12 in China from 2006 to 2018.

The original sequence data in Fig 2 span 156 months, from January 2006 to December 2018, and show a noticeable pattern of increasing scrub typhus cases together with seasonal periodicity. The initial sequence appears to be nonstationary, as seen in Fig 2, indicating that trend differenced, seasonally differenced, or enhanced Dickey-Fuller tests ought to be run. The time series was stationary upon transformation and first-order seasonal differenced with a period of 12 (Fig 3). Parameters *d, D*, and *s* were therefore, correspondingly, 0, 1, and 12. Moreover, the results of the ADF test (t = −3.643, *P* < 0.001) showed that the time series was stationary following transformation and difference.

**Fig 4:**
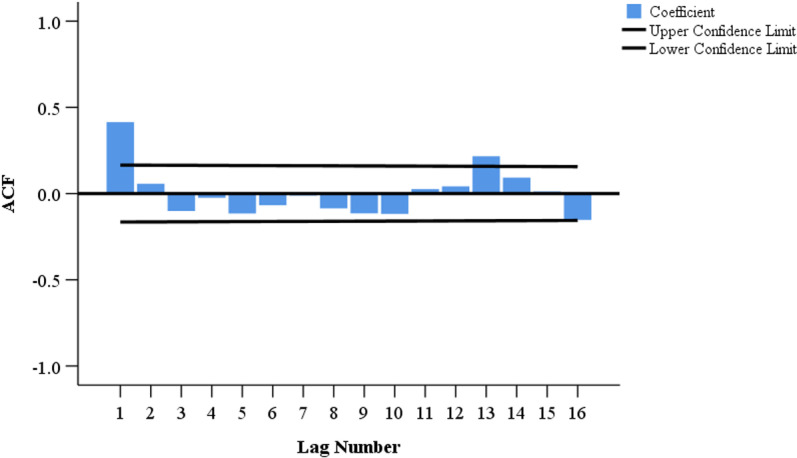
Plot of autocorrelation function (ACF) after a first-order seasonal difference.

**Fig 5:**
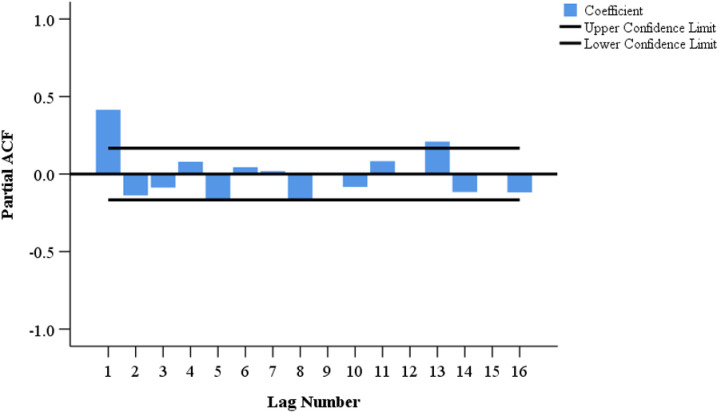
Plot of partial autocorrelation function (PACF) after a first-order seasonal difference.

**Fig 6:**
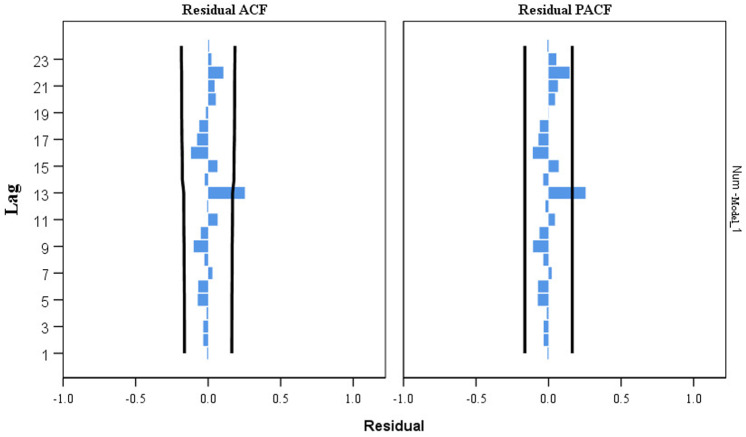
Autocorrelation function (ACF) and partial autocorrelation function (PACF) of the residuals series of the SARIMA (1, 0, 2) (1, 1, 1)_12_ model.

We provisionally selected seven models based on the characteristics of the ACF and PACF distributions (Figs 4 and 5). The most optimal SARIMA (1, 0, 2) (1, 1, 1) _12_ model has been confirmed based on the goodness-of-fit test statistics. This model had the lowest BIC (BIC = 12.041), lowest RMSE (RMSE = 371.268), and comparatively high *R*^2^ (*R*^2^ = 0.919) (S1 File). Table 4 shows the estimated SARIMA model parameters as well as the testing results. Furthermore, the ACF and PACF of the SARIMA residuals (1, 0, 2) (1, 1, 1) _12_ were within the random confidence interval, showing that the residuals did not deviate from a zero-mean white noise process (Fig 6). Finally, the Ljung-Box *Q* test results (*Q *= 19.671, *P* = 0.104) for the model indicates that the residual series was a white noise sequence.

**Table 4:** Goodness of fits for the SARIMA models corresponding to different choices of *p*, *q* and *P*, *Q.*

Model	BIC	*R* ^2^	RMSE	MAPE	Ljung-Box Q statistics	*P* value^*^
SARIMA (1,0,1) (1,1,1)_12_	12.057	0.914	380.700	157.844	32.190	0.004
SARIMA (1,0,2) (1,1,1)_12_	**12.041**	**0.919**	**371.268**	167.592	19.671	**0.104**
SARIMA (1,0,1) (2,1,2)_12_	12.140	0.914	383.446	158.020	32.495	0.001
SARIMA (2,0,1) (2,1,2)_12_	12.160	0.916	380.705	184.415	24.036	0.013
SARIMA (1,0,1) (1,1,2)_12_	12.098	0.914	382.000	156.303	32.533	0.002
SARIMA (2,0,2) (1,1,2)_12_	12.121	0.919	373.236	174.914	21.689	0.027
SARIMA (1,0,1) (2,1,1)_12_	12.098	0.914	382.025	156.917	32.666	0.002

* *P* value calculated by use of Ljung-Box Q test. *P* ≤ 0.05, it means that the model fitting is unsuccessful and needs to be adjusted; until *P* > 0.05, then the residual series is white noise, and the model can be used to make predictions.

**Fig 7:**
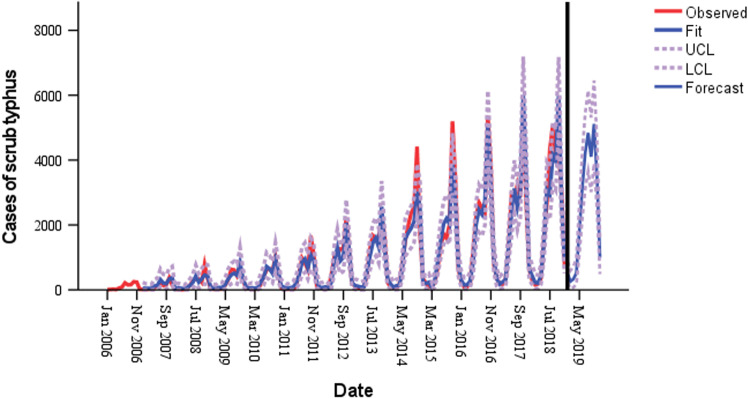
SARIMA (1, 0, 2) (1, 1, 1)_12_ model fitting, verification and forecasting of scrub typhus in China from January 2006 to December 2018 (UCL, upper confidence limit; LCL, low confidence limit).

For the 12 months of 2019—from January to December—the monthly cases of scrub typhus were predicted using the SARIMA (1, 0, 2) (1, 1, 1) _12_ model. Fig 7 shows the values for the number of scrub typhus cases that are predicted by the model and the observed values. The values of observed and predicted in the year of 2019 were displayed in Table 5. Furthermore, the predicted values were within the 95% confidence interval (S2 File).

**Table 5:** Comparison of observed number of scrub typhus cases and corresponding out-of-sample predicted values from January to December in 2019 by the SARIMA (1, 0, 2) (1, 1, 1) _12_ model.

Months	Observed numbers	Predicted numbers	95% *CI*
January	300	505	217 ~ 875
February	212	264	47 ~ 596
March	196	335	77 ~ 712
April	544	521	180 ~ 982
May	1547	1786	1092 ~ 2600
June	2364	3149	2205 ~ 4212
July	2814	4224	3121 ~ 5447
August	3906	4840	3655 ~ 6146
September	3966	4104	3017 ~ 5310
October	5745	5116	3895 ~ 6456
November	4291	3472	2478 ~ 4586
December	634	987	489 ~ 1604
Total	26519	29303	

*CI* is confidence interval.

## Discussion

In mainland China, there were 142849 cases of scrub typhus in 31 provinces (or municipalities) between 2006 and 2018. Our study revealed varying seasonal patterns of the scrub typhus epidemic in China. Scrub typhus showed seasonal characteristics from July to November (S4 Table). These findings were consistent with earlier research reports indicating that the greatest number of cases (86.8%) in Guangzhou occurred between May and October [23]. Since chigger mites are the only vector of scrub typhus. Therefore, the mainly reason for the seasonal patterns of scrub typhus is the seasonal appearance of chigger mite larvae. In China, scrub typhus is mostly spread by *Leptotrombidium deliense* and *L. scutellare* [24]. *L. deliense* inhabits southern China and emerges in April, peaking from June to August, and decreasing from September to December, whereas *L. scutellare* is widespread in China, emerging annually from October to December, and is the main mite species in northern China [25]. The heterogeneous geographic distribution of *L. deliense* and *L. scutellare* may well explain the diverse seasonal patterns of scrub typhus in the southeast, southwest, and middle-east parts of China [25]. The seasonal patterns of scrub typhus in China can be divided into two types, one is the summer type, which is mainly prevalent in the southern part of China, the main animal hosts are *Rattus losea* and *R. tanezumi*, the main vector chigger mites are *Leptotrombidium deliense*, the epidemic period is from June to August, and the type of *Orientia tsutsugamushi* is Gilliam. The other is the fall-winter type, which is mainly prevalent in Jiangsu and Shandong provinces, the main animal hosts are *Apodemus agrarius*, *R*. *norvegicus* and *Tscherskia triton*, the main vector chigger mite is *L. scutellare*, the epidemic period is 10–12, and the type of *Orientia tsutsugamushi* is Kawasaki [26,27]. Undoubtedly, these seasonal patterns variation will prompt public health departments to allocate resources according to the peak of incidence and the months with more reported cases when formulating scrub typhus control policies.

Cases and provinces of scrub typhus rose dramatically every year. 16 provinces and 1244 cases of scrub typhus were reported in 2006. However, in 2018, there were 31 provinces and 26757 cases of scrub typhus (Fig 1). Scrub typhus has spread from the south, southwest, southeast, and eastern coasts to the center, north, northeast, and northwest of mainland China (S2 Table). Every year, scrub typhus spread from the site to the adjacent regions, from south to north. Sporadic cases and disease outbreaks started to be identified in the northern provinces of Jiangsu, Shandong, Beijing, and Tianjin, as well as the emergence of new natural foci in recent decades due to the fast development of society and economy, climate change, population movement, improved recognition by medical professionals, and constantly improving detection techniques [28–30]. Scrub typhus can have a 30% or more case fatality rate if treatment is not received [7]. Scrub typhus vaccinations are still not approved as of now. As a result, mainland China should pay attention to scrub typhus and implement efficient prevention and control measures.

Public health authorities must accurately predict and estimate infectious diseases in order to fully comprehend their epidemic characteristics and implement preventive and control measures ahead of time [31,32]. Scrub typhus is an infectious illness that occurs in various parts of the world [5,33]. The use of historical data to predict future scrub typhus cases has lately gained importance in public health research due to the background of big data. Consequently, it is crucial to study the frequency of scrub typhus infections using predictive models. It is crucial for both preventing and managing infections caused by scrub typhus. These mathematical models have been employed in numerous research in recent years to forecast the spread of infectious diseases, including TB, mumps, dengue, malaria, and, most recently, COVID-19 [34–37].

Autoregressive Integrated Moving Average (ARIMA) and its variants, such as Seasonal Autoregressive Integrated Moving Average (SARIMA), were commonly employed for future forecasts. Temporal models, such as ARIMA, are useful for short-term forecasting because they can effectively capture time series patterns, particularly when trends and seasonality are present. However, ARIMA cannot handle abrupt shifts since it has trouble with irregular data and needs long, stable time-series data to be accurate. SARIMA enhances this by managing seasonal variations, which is helpful for climatically cycle-influenced diseases like scrub typhus [38,39]. Our study revealed that the SARIMA (1, 0, 2) (1, 1, 1) _12_ model was the most accurate mathematical model for predicting monthly scrub typhus cases in China, based on surveillance data from January 2006 to December 2018. After that, the model was used to forecast the number of scrub typhus cases every month for a 12-month period in 2019 from January to December. The findings demonstrated that the model has a respectable level of accuracy in forecasting scrub typhus cases in China, with the actual monthly cases falling within 95% *CI* of the predicted values and the RMSE and MAPE between the predicted and actual values being 371.268 and 167.592, respectively. The predicted value in 2019 differed slightly from the actual cases, but the changing trends of the two were essentially the same. Because infectious disease cases are influenced by various factors, the time series model cannot predict them perfectly, but the model’s predicted value can nevertheless reliably estimate future increases. Precise forecasting of the scrub typhus cases in this paper can help local health administrative departments release an early warning of the increased risk of scrub typhus incidence and distribute reasonable medical resources in a timely manner for preventing and controlling the scrub typhus spread.

Our research is original and essential since the emergence of developing scrub typhus outbreaks in China has had a substantial impact on public health [19,40–42]. To the best of our knowledge, this work is the first to predict future cases of scrub typhus using SARIMA modeling. When understanding the forecasting findings, it is important to note some of this model’s limitations. First of all, the data came from the China Center for Disease Control and Prevention, a passive surveillance system. Because subclinical or asymptomatic cases of scrub typhus that did not seek medical attention were not entered into the system, the reported data may have underestimated the number of cases and could affect the precision of the predictions. Geographic comparisons of incidence may be impacted by regional differences in diagnosis and case notification. We were unable to present the complete picture of scrub typhus in China, including human cases, infections, hosts, and vectors, since we lacked data on *O. tsutsugamushi* strains and the precise distribution of *Leptotrombidium* mite species. The data utilized in this study, however, were the most thorough and trustworthy data on scrub typhus that were available in China at the national and subnational levels; these nationwide report data show notable shifts in the epidemiologic characteristics of scrub typhus in China, underscoring the necessity of carrying out additional high-caliber research to more accurately interpret the results from passive surveillance data. Second, the SARIMA model is only suitable for short-term forecasting, and it must be dynamically updated with new data in order to assure forecast accuracy and stability. Third, it is crucial to note that environmental, climatic, and socioeconomic factors may influence scrub typhus outbreaks [42]; however, we were unable to include these information in our analysis. The constant surveillance of scrub typhus is critical for its control since it allows for the evaluation of the past and present in order to lessen the future burden of this essential neglected tropical disease. More importantly, this study highlights the importance of taking steps to reduce underreporting.

## Supporting information

S1 File The output results of different model statistics. (PDF)

S1 Table Epidemiologic features of scrub typhus. (XLSX)

S2 File The output results of 95% CI. (PDF)

S2 Table Reported cases of scrub typhus in 31 provinces. (XLSX)

S3 Table Sex, age and geographic area characteristics. (XLSX)

S4 Table Number of scrub typhus cases per month 2006–2019. (XLSX)

S5 Table The minimal data set. (XLSX)
