# Supplementary material for: A Seasonal Autoregressive Integrated Moving Average (SARIMA) forecasting model to predict the epidemic trends of scrub typhus in China
Source: PLoS One. 2025 Jun 23;20(6):e0325905. doi: 10.1371/journal.pone.0325905 (PMC12184917; doi:10.1371/journal.pone.0325905)
Supplement: S1 File — (PDF) [file pone.0325905.s001.pdf]

SARIMA (1, 0, 2) (1, 1, 1) $_{12}$ model （The most optimal model）

Model Fit

Fit Statistic	Mean	SE	Minimum	Maximum	Percentile						
					5	10	25	50	75	90	95
Stationary R-squared	.238	.	.238	.238	.238	.238	.238	.238	.238	.238	.238
R-squared	.919	.	.919	.919	.919	.919	.919	.919	.919	.919	.919
RMSE	371.268	.	371.268	371.268	371.268	371.268	371.268	371.268	371.268	371.268	371.268
MAPE	167.592	.	167.592	167.592	167.592	167.592	167.592	167.592	167.592	167.592	167.592
MaxAPE	5465.372	.	5465.372	5465.372	5465.372	5465.372	5465.372	5465.372	5465.372	5465.372	5465.372
MAE	238.304	.	238.304	238.304	238.304	238.304	238.304	238.304	238.304	238.304	238.304
MaxAE	1627.086	.	1627.086	1627.086	1627.086	1627.086	1627.086	1627.086	1627.086	1627.086	1627.086
Normalized BIC	12.041	.	12.041	12.041	12.041	12.041	12.041	12.041	12.041	12.041	12.041

Model Statistics

Model	Number of Predictors	Model Fit statistics		Ljung-Box Q(18)			Number of Outliers
		Stationary R-squared	R-squared	Statistics	DF	Sig.	
Num-模型_1	0	.238	.919	19.671	13	.104	0

SARIMA (1,0,1) $(1,1,1)_{12}$ model

Model Description

			Model Type
Model ID	Num	模型_1	ARIMA(1,0,1)(1,1,1)

Model Summary

Model Fit

Fit Statistic	Mean	SE	Minimum	Maximum	Percentile 5	10	25	50	75	90	95
Stationary R-squared	.193	.	.193	.193	.193	.193	.193	.193	.193	.193	.193
R-squared	.914	.	.914	.914	.914	.914	.914	.914	.914	.914	.914
RMSE	380.700	.	380.700	380.700	380.700	380.700	380.700	380.700	380.700	380.700	380.700
MAPE	157.844	.	157.844	157.844	157.844	157.844	157.844	157.844	157.844	157.844	157.844
MaxAPE	4066.807	.	4066.807	4066.807	4066.807	4066.807	4066.807	4066.807	4066.807	4066.807	4066.807
MAE	236.032	.	236.032	236.032	236.032	236.032	236.032	236.032	236.032	236.032	236.032
MaxAE	1633.854	.	1633.854	1633.854	1633.854	1633.854	1633.854	1633.854	1633.854	1633.854	1633.854
Normalized BIC	12.057	.	12.057	12.057	12.057	12.057	12.057	12.057	12.057	12.057	12.057

Model Statistics

Model	Number of Predictors	Model Fit statistics		Ljung-Box Q(18)			Number of Outliers
		Stationary R-squared	R-squared	Statistics	DF	Sig.	
Num-模型_1	0	.193	.914	32.190	14	.004	0

SARIMA (1,0,1) $(2,1,2)_{12}$ model

Model Description

			Model Type
Model ID	Num	模型_1	ARIMA(1,0,1)(2,1,2)

Model Summary

Model Fit

Fit Statistic	Mean	SE	Minimum	Maximum	Percentile 5	10	25	50	75	90	95
Stationary R-squared	.193	.	.193	.193	.193	.193	.193	.193	.193	.193	.193
R-squared	.914	.	.914	.914	.914	.914	.914	.914	.914	.914	.914
RMSE	383.446	.	383.446	383.446	383.446	383.446	383.446	383.446	383.446	383.446	383.446
MAPE	158.020	.	158.020	158.020	158.020	158.020	158.020	158.020	158.020	158.020	158.020
MaxAPE	4077.810	.	4077.810	4077.810	4077.810	4077.810	4077.810	4077.810	4077.810	4077.810	4077.810
MAE	236.052	.	236.052	236.052	236.052	236.052	236.052	236.052	236.052	236.052	236.052
MaxAE	1634.417	.	1634.417	1634.417	1634.417	1634.417	1634.417	1634.417	1634.417	1634.417	1634.417
Normalized BIC	12.140	.	12.140	12.140	12.140	12.140	12.140	12.140	12.140	12.140	12.140

Model Statistics

Model	Number of Predictors	Model Fit statistics: Stationary R-squared	R-squared	Ljung-Box Q(18): Statistics	DF	Sig.	Number of Outliers
Num-模型_1	0	.193	.914	32.495	12	.001	0

SARIMA (2,0,1) $(2,1,2)_{12}$ model

Model Description

			Model Type
Model ID	Num	模型_1	ARIMA(2,0,1)(2,1,2)

Model Summary

Model Fit

Fit Statistic	Mean	SE	Minimum	Maximum	Percentile						
					5	10	25	50	75	90	95
Stationary R-squared	.210	.	.210	.210	.210	.210	.210	.210	.210	.210	.210
R-squared	.916	.	.916	.916	.916	.916	.916	.916	.916	.916	.916
RMSE	380.705	.	380.705	380.705	380.705	380.705	380.705	380.705	380.705	380.705	380.705
MAPE	184.415	.	184.415	184.415	184.415	184.415	184.415	184.415	184.415	184.415	184.415
MaxAPE	5592.830	.	5592.830	5592.830	5592.830	5592.830	5592.830	5592.830	5592.830	5592.830	5592.830
MAE	240.148	.	240.148	240.148	240.148	240.148	240.148	240.148	240.148	240.148	240.148
MaxAE	1666.885	.	1666.885	1666.885	1666.885	1666.885	1666.885	1666.885	1666.885	1666.885	1666.885
Normalized BIC	12.160	.	12.160	12.160	12.160	12.160	12.160	12.160	12.160	12.160	12.160

Model Statistics

Model	Number of Predictors	Model Fit statistics		Ljung-Box Q(18)			Number of Outliers
		Stationary R-squared	R-squared	Statistics	DF	Sig.	
Num-模型_1	0	.210	.916	24.036	11	.013	0

SARIMA (1,0,1) (1,1,2)$_{12}$ model

Model Description

			Model Type
Model ID	Num	模型_1	ARIMA(1,0,1)(1,1,2)

Model Summary

Model Fit

Fit Statistic	Mean	SE	Minimum	Maximum	Percentile						
					5	10	25	50	75	90	95
Stationary R-squared	.193	.	.193	.193	.193	.193	.193	.193	.193	.193	.193
R-squared	.914	.	.914	.914	.914	.914	.914	.914	.914	.914	.914
RMSE	382.000	.	382.000	382.000	382.000	382.000	382.000	382.000	382.000	382.000	382.000
MAPE	156.303	.	156.303	156.303	156.303	156.303	156.303	156.303	156.303	156.303	156.303
MaxAPE	4064.213	.	4064.213	4064.213	4064.213	4064.213	4064.213	4064.213	4064.213	4064.213	4064.213
MAE	235.316	.	235.316	235.316	235.316	235.316	235.316	235.316	235.316	235.316	235.316
MaxAE	1629.831	.	1629.831	1629.831	1629.831	1629.831	1629.831	1629.831	1629.831	1629.831	1629.831
Normalized BIC	12.098	.	12.098	12.098	12.098	12.098	12.098	12.098	12.098	12.098	12.098

Model Statistics

Model	Number of Predictors	Model Fit statistics		Ljung-Box Q(18)			Number of Outliers
		Stationary R-squared	R-squared	Statistics	DF	Sig.	
Num-模型_1	0	.193	.914	32.533	13	.002	0

SARIMA (2,0,2) $(1,1,2)_{12}$ model

Model Description

			Model Type
Model ID	Num	模型_1	ARIMA(2,0,2)(1,1,2)

Model Summary

Model Fit

Fit Statistic	Mean	SE	Minimum	Maximum	Percentile 5	10	25	50	75	90	95
Stationary R-squared	.241	.	.241	.241	.241	.241	.241	.241	.241	.241	.241
R-squared	.919	.	.919	.919	.919	.919	.919	.919	.919	.919	.919
RMSE	373.236	.	373.236	373.236	373.236	373.236	373.236	373.236	373.236	373.236	373.236
MAPE	174.914	.	174.914	174.914	174.914	174.914	174.914	174.914	174.914	174.914	174.914
MaxAPE	5902.033	.	5902.033	5902.033	5902.033	5902.033	5902.033	5902.033	5902.033	5902.033	5902.033
MAE	239.803	.	239.803	239.803	239.803	239.803	239.803	239.803	239.803	239.803	239.803
MaxAE	1672.867	.	1672.867	1672.867	1672.867	1672.867	1672.867	1672.867	1672.867	1672.867	1672.867
Normalized BIC	12.121	.	12.121	12.121	12.121	12.121	12.121	12.121	12.121	12.121	12.121

Model Statistics

Model	Number of Predictors	Model Fit statistics: Stationary R-squared	R-squared	Ljung-Box Q(18): Statistics	DF	Sig.	Number of Outliers
Num-模型_1	0	.241	.919	21.689	11	.027	0

SARIMA (1,0,1) $(2,1,1)_{12}$ model

Model Description

			Model Type
Model ID	Num	模型_1	ARIMA(1,0,1)(2,1,1)

Model Summary

Model Fit

Fit Statistic	Mean	SE	Minimum	Maximum	Percentile						
					5	10	25	50	75	90	95
Stationary R-squared	.193	.	.193	.193	.193	.193	.193	.193	.193	.193	.193
R-squared	.914	.	.914	.914	.914	.914	.914	.914	.914	.914	.914
RMSE	382.025	.	382.025	382.025	382.025	382.025	382.025	382.025	382.025	382.025	382.025
MAPE	156.917	.	156.917	156.917	156.917	156.917	156.917	156.917	156.917	156.917	156.917
MaxAPE	4067.112	.	4067.112	4067.112	4067.112	4067.112	4067.112	4067.112	4067.112	4067.112	4067.112
MAE	235.590	.	235.590	235.590	235.590	235.590	235.590	235.590	235.590	235.590	235.590
MaxAE	1632.567	.	1632.567	1632.567	1632.567	1632.567	1632.567	1632.567	1632.567	1632.567	1632.567
Normalized BIC	12.098	.	12.098	12.098	12.098	12.098	12.098	12.098	12.098	12.098	12.098

Model Statistics

Model	Number of Predictors	Model Fit statistics		Ljung-Box Q(18)			Number of Outliers
		Stationary R-squared	R-squared	Statistics	DF	Sig.	
Num-模型_1	0	.193	.914	32.666	13	.002	0
