# Supplementary material for: A Seasonal Autoregressive Integrated Moving Average (SARIMA) forecasting model to predict the epidemic trends of scrub typhus in China
Source: PLoS One. 2025 Jun 23;20(6):e0325905. doi: 10.1371/journal.pone.0325905 (PMC12184917; doi:10.1371/journal.pone.0325905)
Supplement: S2 File — (PDF) [file pone.0325905.s003.pdf]

Model Statistics

Model	Number of Predictors	Model Fit statistics	Ljung-Box Q(18)			Number of Outliers
		Stationary R-squared	Statistics	DF	Sig.	
Number-模型_1	0	.306	24.953	13	.023	0

Forecast

Model		Jan 2019	Feb 2019	Mar 2019	Apr 2019	May 2019	Jun 2019	Jul 2019	Aug 2019	Sep 2019	Oct 2019	Nov 2019	Dec 2019
Number-模型_1	Forecast	505	264	335	521	1786	3149	4224	4840	4104	5116	3472	987
	UCL	875	596	712	982	2600	4212	5447	6146	5310	6456	4586	1604
	LCL	217	47	77	180	1092	2205	3121	3655	3017	3895	2478	489

For each model, forecasts start after the last non-missing in the range of the requested estimation period, and end at the last period for which non-missing values of all the predictors are available or at the end date of the requested forecast period, whichever is earlier.
